# Fatal outcome of severe serotonin syndrome and acute respiratory distress syndrome after self-poisoning with paroxetine: a case report

**DOI:** 10.1186/s13256-025-05602-7

**Published:** 2025-10-13

**Authors:** Sina M. Pütz, Christoph Hüser, Oezguer A. Onur, David Steindl, Dennis A. Eichenauer, Jan-Hendrik Naendrup, Marie Anne-Catherine Neumann, Nathalie M. Schmidt, Alexander F.vom Stein, Boris Böll

**Affiliations:** 1 Department I of Internal Medicine, Division of Infectious Diseases, University of Cologne, Faculty of Medicine and University Hospital Cologne, Kerpener Straße 62, 50937 Cologne, Germany; 2 Emergency Department, University of Cologne, Faculty of Medicine and University Hospital Cologne, Cologne, Germany; 3 Department of Neurology, University Hospital Cologne, Cologne, Germany; 4 Poison Control Center, Charité–Universitätsmedizin Berlin, Charitéplatz 1, 10117 Berlin, Germany; 5 Department I of Internal Medicine, Center for Integrated Oncology Aachen Bonn Cologne Duesseldorf (CIO ABCD), University of Cologne, Faculty of Medicine and University Hospital of Cologne, Cologne, Germany; 6 Institute for Metabolomics in Ageing, Cluster of Excellence Cellular Stress Responses in Aging-Associated Diseases (CECAD), University of Cologne, Faculty of Medicine and University Hospital Cologne, Cologne, Germany

**Keywords:** Paroxetine, Case report, Serotonin syndrome, Acute respiratory distress syndrome, Hemoadsorption

## Abstract

**Background:**

Paroxetine overdose can cause serotonin syndrome, presenting with a constellation of manifestations that range from mild symptoms to life-threatening complications. Current evidence regarding the management of paroxetine-induced serotonin syndrome is limited to case reports and small case series, with no definitive recommendations established from controlled clinical trials in humans.

**Case presentation:**

In this case report, we describe an exceptional and severe clinical course of a 26-year-old Caucasian female who ingested multiple drugs with suicidal intention—including 4000 mg paroxetine. The patient developed severe serotonin syndrome complicated by acute respiratory distress syndrome, necessitating invasive mechanical ventilation and veno-venous extracorporeal membrane oxygenation. Serotonin syndrome prevailed unusually long, with serum paroxetine levels remaining within the lethal dose range for 20 days postingestion. Despite intensive therapeutic interventions—including CytoSorb™ hemoadsorption, adjunctive corticosteroids, olanzapine (10 mg/day), and maximal intensive care treatment—the patient died on day 20 postingestion.

**Conclusion:**

Our case illustrates the importance of early and aggressive intervention, while highlighting the limited efficacy of currently available extracorporeal elimination techniques in severe paroxetine overdose. Given the protracted and progressive symptoms observed in this case report and others, clinicians should remain vigilant for pharmacokinetic complications such as pharmacobezoars or poor CYP2D6 metabolism in massive selective serotonin reuptake inhibitor overdose, leading to an early and aggressive treatment in similar cases with serotonin syndrome.

**Supplementary Information:**

The online version contains supplementary material available at 10.1186/s13256-025-05602-7.

## Background

Paroxetine is a potent and selective serotonin reuptake inhibitor (SSRI) approved for treating depression, panic disorders, obsessive–compulsive disorders, and anxiety disorders. An overdose can cause a serotonin syndrome, mostly presenting with mild symptoms such as tachycardia, hyperthermia, and hyperreflexia or more severe symptoms including fever, delirium, and coma [1, 2]. Most cases of SSRI poisoning are mild and treatment is typically supportive [1, 3], but there are few case reports describing the clinical course and management of serotonin syndrome resulting from primarily unintentional paroxetine overdose [4–8]. Here, we describe a rare and severe case of serotonin syndrome complicated by acute respiratory distress syndrome (ARDS) owing to multidrug poisoning with prolonged paroxetine toxicity. The therapeutic approach involved veno-venous extracorporeal membrane oxygenation (vv-ECMO) and CytoSorb™ hemadsorption—highlighting the clinical complexity and therapeutic challenges of managing severe serotonin syndrome.

## Case presentation

**Fig. 1:**
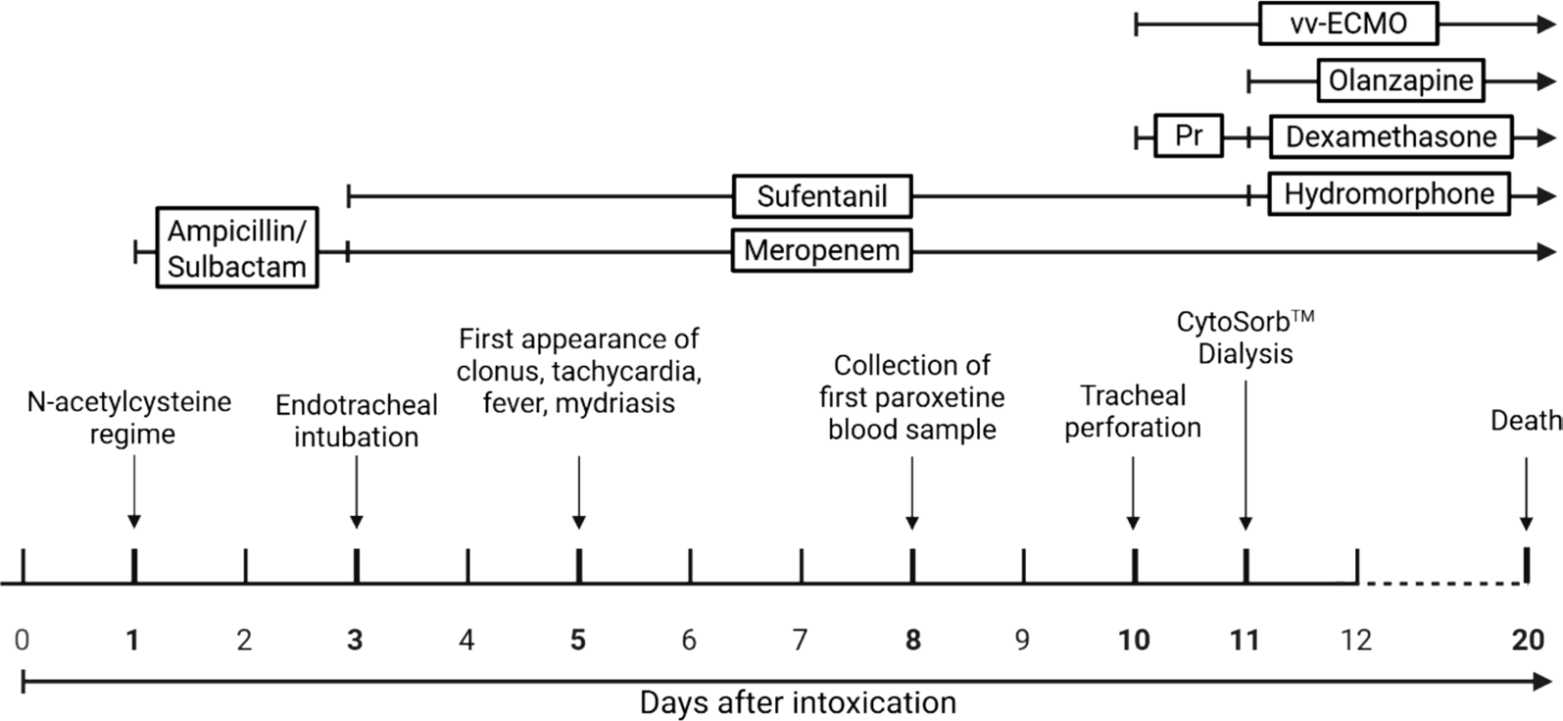
Course of therapy from ingestion to the patient’s death in days. *vv-ECMO* veno-venous extracorporeal membrane oxygenation, *Pr* Prednisolone. Figure created with Biorender

**Fig. 2:**
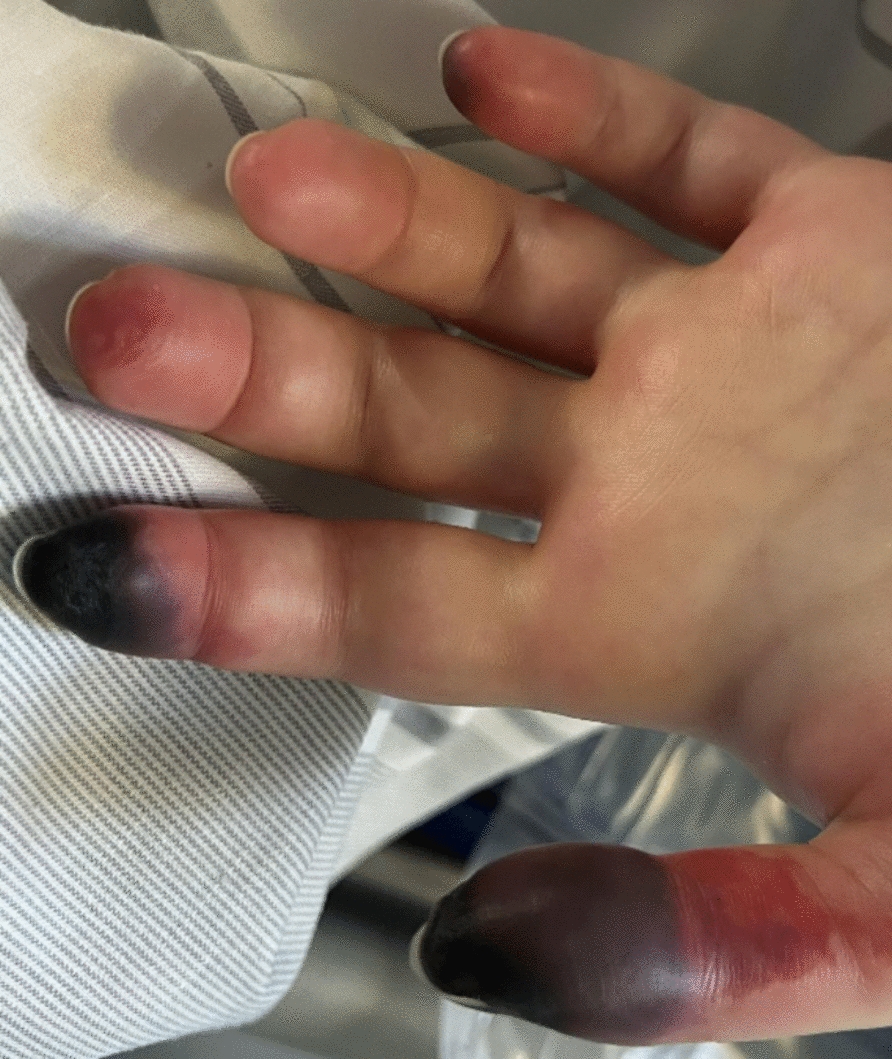
Acral necrosis of the left hand

**Fig. 3:**
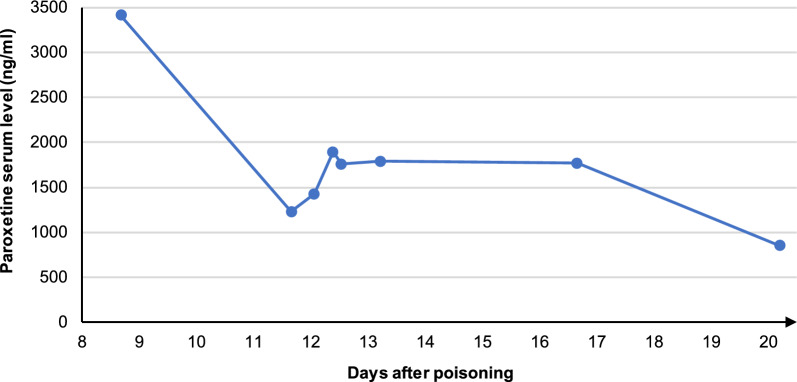
Course of paroxetine serum level after intoxication with 4000 mg paroxetine on day 0

A 26-year-old Caucasian woman with an estimated body weight of 50 kg ingested a total amount of 3150 mg agomelatine (63 mg/kg), 4000 mg paroxetine (80 mg/kg), 8000 mg paracetamol (160 mg/kg), 5000 mg acetylsalicylic acid (100 mg/kg), 1000 mg caffeine (20 mg/kg), and 25,200 mg ibuprofen (504 mg/kg) in suicidal intent (total of 336 pills). The patient had a history of social phobia since childhood and a diagnosis of recurrent depressive disorder. The patient was reported to be on regular paroxetine therapy. However, the exact dosage and frequency of intake were unknown. On day 1 postingestion, the patient was admitted to a local hospital by ambulance in a somnolent and disoriented condition, with a Glasgow coma scale score of 12. Vital signs included a heart rate of 140 beats per minute, blood pressure of 125/75 mmHg, respiratory rate of 14 breaths per minute, and a temperature of 36 °C. On admission, physical examination revealed clear and rhythmic heart sounds. Pulmonary auscultation demonstrated vesicular breath sounds with symmetrical ventilation bilaterally. The electrocardiogram showed a tachycardic sinus rhythm with an intermediate axis and no repolarization abnormalities. Focused transthoracic echocardiography demonstrated good left and right ventricular systolic function without regional wall motion abnormalities. No valvular pathology or pericardial effusion was detected. Owing to reported paracetamol overdose, N-acetylcysteine was administered (Fig. 1) according to the regimen described by Prescott *et al*. [9]. All other medication—except for paroxetine—was deemed to be no longer clinically relevant by the contacted poison information center, based on the time elapsed since ingestion. After treatment with acetylcysteine, no clinically relevant paracetamol poisoning (e.g. elevated transaminases) developed (Table 1). However, on day 2, an acute respiratory deterioration occurred with signs of pneumonia visible on the chest x-ray. The patient was put on noninvasive ventilation and subsequently required mechanical ventilation on day 3 owing to respiratory failure. ARDS Berlin criteria [10] were fulfilled with (1) an onset within 1 week, (2) the described results of the chest x-ray, (3) respiratory failure not explained by cardiac failure or fluid overload, and (4) an impaired oxygenation with a PaO_2_/FiO_2_ ratio of 70 mmHg, resulting in a stage of severe ARDS. The patient was then transferred to our tertiary care institution for specialized ARDS treatment. Bronchoalveolar lavage results showed no microbiological or virological pathogens. ARDS was managed by prone positioning and airway pressure release ventilation and the respiratory condition improved, allowing for initiation of weaning and reduction of sedatives on day 4. After reducing sedation sufentanil and midazolam, the patient developed pathological delirium and agitation. Moreover, an inducible clonus—primarily of the lower extremities—was observed. This warranted neurological evaluation and cerebral imaging: tibial nerve somatosensory evoked potentials (SEP) showed an involvement of the sensory pathways of both legs, while further comprehensive neurological diagnostic testing with cerebral computed tomography (CT) and magnetic resonance imaging (MRI), cerebrospinal fluid puncture, electroencephalography (EEG), and SEP of the median nerve did not show pathological findings. Furthermore, an increasing livid discoloration of the acra (Fig. 2), mydriasis, tachycardia up to 142 bpm, hypotension up to 82/52 mmHg—necessitating the use of catecholamines—and recurrent fever up to 39.5 °C occurred (Table 2). Taken together with inducible clonus, a serotonin syndrome due to paroxetine overdose was suspected. This diagnosis was supported by positive Hunter Serotonin Toxicity Criteria [11]. As no severe life-threatening serotonin syndrome symptoms were present at that point and the patient´s condition kept improving, the decision for supportive treatment was made. In parallel, efforts were made to make the serotonin antagonist cyproheptadine readily available in case of progressing symptoms; however, it was not available in Germany. A paroxetine level of day 8 was markedly elevated with 3414 ng/mL (indicated values of the test laboratory: therapeutic range 20–65 ng/mL, critical value > 120 ng/mL) and the patient started deteriorating on day 10 (Table 2). Therefore, a symptomatic therapy with olanzapine (10 mg/day) was started. Analgosedation with sufentanil was switched to hydromorphone to prevent further aggregation of serotonin, and corticosteroids were administered in an attempt to enhance paroxetine elimination by CYP2D6 induction. To complicate matters, a tracheal perforation occurred when a tracheal cannula was inserted on day 10. Following thorough interdisciplinary consultation, it was concluded that a conservative approach with traction granulation of the extensive tracheal defect represented the most effective therapeutic approach. However, elevated ventilatory pressures were anticipated to impair wound healing. To mitigate this risk and reduce the need for high-pressure invasive ventilation, vv-ECMO was necessary. Once vv-ECMO was initiated, CytoSorb™ hemoadsorption could easily be added in an attempt to eliminate paroxetine. Paroxetine levels were monitored, but did not indicate any relevant adsorption by CytoSorb™ (Fig. 3**)**. Unfortunately, the patient continued to deteriorate with complete dependence on vv-ECMO for gas exchange, severe bipulmonary opacities on chest CT, a reliance on high dose vasopressors, and a persistent high paroxetine level. With limits of available treatment options, the therapeutic objective was revised to best supportive care on day 20. The patient subsequently died receiving sufficient symptomatic management.

**Table 1:** History of relevant laboratory values starting from day 4, when the patient was transferred to the tertiary care hospital, until death on day 20

Day after ingestion	CRP (mg/L)	PCT (µg/L)	Creatinine (mg/dL)	Bilirubin (mg/dL)	AST (U/L)	INR
4	425.5	6.1	0.59	0.6	48	1.2
5	410.0	4.1	0.47	0.5	175	1.3
6	414.3	24.4	0.62	0.7	440	1.4
7	369.2	37.4	0.64	0.6	455	1.4
8	327.3	18.6	0.55	0.4	280	1.6
9	314.7	8.8	0.50	0.2	216	1.4
10	225.4	5.8	0.55	0.5	136	1.2
11	250.4	8.3	0.43	0.5	239	1.3
12	224.5	2.2	0.29	0.8	203	1.2
13	233.9	2.6	0.46	0.7	196	1.4
14	217.1	1.4	0.33	1.2	187	1.1
15	255.4	n.m	0.29	1.4	183	1.0
16	276.1	n.m	0.26	1.5	172	1.0
17	330.1	n.m	0.24	1.5	160	1.0
18	311.0	0.7	0.28	1.4	117	1.0
19	348.4	n.m	0.30	1.4	115	1.1
20	446.1	0.7	0.33	2.1	97	1.1

*CRP* C-reactive protein, *PCT* procalcitonin, *AST* aspartate aminotransferase, *INR* international normalized ratio, *n.m.* not measured

**Table 2:** History of arterial blood gas analyses, vital signs, oxygenation, and catecholamine infusion rates starting from day 4, when the patient was transferred to the tertiary care hospital, until death on day 20

Day after ingestion	highest pCO_2_ (mmHg)	lowest pO_2_ (mmHg)	FiO_2_ range (%)	Lowest SpO_2_ (%)	Highest body temperature (°C)	Highest rate noradrenaline (µg/kg/min)
4	44.0	73.1	35–45	93	39.2	0.15
5	52.1	71.6	30–55	84	39.0	0.10
6	59.7	67.3	35–60	90	39.1	0.10
7	61.3	55.7	35–70	87	39.5	0.17
8	69.5	72.9	45–70	86	39.1	0.14
9	69.4	79.0	50–60	76	38.4	0.14
10	105.0	41.8	45–100	26	38.0	0.60
11	52.2	51.7	40–40	83	37.0	0.17
12	59.6	63.2	40–40	40	36.8	0.04
13	67.0	54.8	40–60	65	37.2	0.01
14	59.8	52.6	50–80	68	37.1	0.01
15	55.4	53.1	50–65	83	37.1	0.01
16	66.7	53.8	50–60	74	36.3	0.01
17	65.8	48.0	50–100	68	36.7	0.06
18	56.9	72.1	40–50	90	36.2	0.09
19	56.6	65.5	40–40	89	36.1	0.12
20	46.5	62.9	40–100	90	36.3	0.30

Note that veno-venous extracorporeal membrane oxygenation therapy was started on day 10

*pCO*_*2*_ Partial pressure of carbon dioxide, *pO*_*2*_ Partial pressure of oxygen, *FiO*_*2*_ Fraction of inspired oxygen, *SpO*_*2*_ Peripheral capillary oxygen saturation

## Discussion

For paroxetine poisoning either in single or multidrug exposure, only few case reports describe severe or fatal serotonin syndrome [4–6], with blood levels ranging from 1400 ng/mL to 3400 ng/mL reported as the lethal threshold [12]. This case report highlights an unusually severe course of paroxetine poisoning with blood levels in the lethal area for weeks as well as the occurrence of ARDS. While the patient had consumed a variety of drugs, the observed course was attributable to paroxetine alone, which was affirmed in repetitive consultations of poison information center experts as well as continuously high paroxetine levels.

Serotonin syndrome typically arises within hours [2] and agonism of 5-HT_2_A-receptors seems to play a critical role in the pathogenesis. The condition occurs less frequently in cases with co-ingestion of 5-HT_2_A-antagonists such as olanzapine [13] and the first-generation antihistamine cyproheptadine, which has higher 5-HT_2_A-antagonistic properties than olanzapine. Both have been proposed and used to treat severe cases of serotonin syndrome. Despite the limited evidence—mostly from case reports and series, with no definitive data from controlled human studies [2]—both agents are typically reserved for severe serotonin syndrome. Since cyproheptadine was unavailable and the patient initially showed mild symptoms with signs of recovery, olanzapine was not administered.

In our case, serotonin syndrome developed on day 5 after ingestion and persisted until the patient’s death on day 20. There are several possible explanations for the delayed onset: (1) the coingestion of agomelatine, which has 5-HT_2_C antagonistic properties, may have initially counteracted the development of serotonin syndrome. After its elimination, paroxetine could have exerted its full serotonergic agonism, leading to the delayed symptom onset. (2) Delayed toxicity may have resulted from a pharmacobezoar, which then resolved after several days. Pharmacobezoars are known to occur not only with certain extended release formulations, such as extended release quetiapine, but also occur after ingestion of large amounts (typically > 75) of pills of different medications [14]. Gastroscopy and administration of activated charcoal were considered on days 1 and 5 but not pursued owing to initial clinical improvement. In retrospect, we would recommend considering such interventions to safely exclude the presence of a pharmacobezoar.

Serotonin syndrome prevailed unusually long, with paroxetine levels in the lethal dose range [12] for 20 days. While paroxetine’s therapeutic half-life is 7–37 h [15], previous case reports documented prolonged half-lives of 8 and 9 days in cases of poisoning with a dose of 2 g and 1 g of paroxetine, respectively [16, 17]. Also, delayed and progressively worsening serotonin toxicity was reported in one previous case report [18]. Since paroxetine blood levels were not measured from day 1, the exact peak concentration is unknown. Therefore, the paroxetine half-life could not be accurately determined in this case. However, as the other ingested medications had likely already been eliminated after time or are not known to interact with paroxetine metabolism, and given that laboratory assessments revealed no evidence of clinically relevant liver injury, a possible explanation for impaired clearance could be that the patient was a CYP2D6-poor-metabolizer. Alternatively, phenoconversion could explain the observation, in which enzymatic activity is functionally reduced despite a normal CYP2D6 genotype. This could result from high paroxetine blood levels causing auto-inhibition, concurrent use of CYP2D6 inhibitors—though absent here—or disease-related alterations affecting enzyme function. Given the lack of CYP2D6 genotyping data and the known autoinhibitory effect of paroxetine on CYP2D6, we cannot clearly distinguish whether the impaired clearance is primarily due to an inherited status as a CYP2D6-poor-metabolizer or to a phenotype conversion owing to high drug exposure or other clinical factors such as an acute inflammation.

Upon receiving the results of the first paroxetine blood level collected on day 8 with extremely elevated levels, and a decision to start aggressive treatment of the serotonin syndrome was made with initiation of olanzapine, which was readily available and has been previously used to treat serotonin syndrome [2, 13]. Alternative atypical antipsychotics were risperidone, clozapine, or chlorpromazine, but we decided on olanzapine as clinical experience is most established with this drug. Moreover, sedation with sufentanil was discontinued and replaced by hydromorphone as a precautionary measure. Although sufentanil is not commonly associated with serotonin syndrome and lacks supporting clinical reports, regulatory warnings and experimental data suggest a potential contribution to serotonergic effects. In the context of our patient’s severe serotonin toxicity, minimizing all possible serotonergic stimuli was considered appropriate [19, 20]. Furthermore, we attempted to induce CYP2D6 using corticosteroids, despite the scarcity of peer-reviewed literature on this subject [21]. The further rationale for corticosteroid administration was their potential to modulate inflammatory responses, which is particularly relevant in the management of ARDS. In addition, the use of an extracorporeal elimination was discussed, but initially discarded after careful risk–benefit assessment. Given paroxetine’s high volume of distribution of 17 L/kg and 95% plasma protein binding at therapeutic doses, both hemodialysis and hemadsorption were judged to be likely ineffective and unnecessarily invasive [15]. However, when the patient required vv-ECMO due to tracheal injury, the risk–benefit assessment changed, as a CytoSorb™ filter for hemadsorption could be easily added to the vv-ECMO circuit. No formal guidelines currently recommend the use of CytoSorb™; its application in this case was experimental and considered as a therapeutic approach [22]. A 6-h CytoSorb™ treatment did not reduce paroxetine levels or lead to clinical improvement. However, as only total blood concentrations were measured without pre- and postadsorber sampling, the device’s elimination capacity could not be assessed.

The patient´s death was ultimately attributed to ARDS. The likelihood of aspiration pneumonia as underlying etiology was considered low, as repeated bronchoalveolar lavage examinations and blood cultures failed to detect any causative pathogens. Nevertheless, given the severity of the clinical course, aspiration pneumonia resulting from the initial reduction in consciousness could not be definitively excluded despite negative microbiological findings. As a result, anti-infective therapy was continued, and upon clinical deterioration on day 3, empiric treatment was switched to meropenem. Furthermore, prior antimicrobial therapy may have impacted microbiological yield and potentially masked an infectious process. Transfusion-related acute lung injury (TRALI) was excluded as a cause, given the absence of prior blood product administration. However, ARDS could be a consequence of paroxetine poisoning itself as severe ARDS was described as a complication of paroxetine poisoning in a previous case report [23] and one patient showed recurrent ARDS with therapeutic paroxetine intake, which stopped after paroxetine discontinuation [24]. Another case report described tremors, muscle rigidity, and sustained ankle clonus, followed by ARDS after intentional overdose of venlafaxine [25]. Nevertheless, in our case, the decision to initiate vv-ECMO therapy was primarily driven by the complication of a tracheal perforation. According to the Extracorporeal Life Support Organization (ELSO) guideline, a large bronchopleural fistula constitutes an indication for vv-ECMO therapy [26]. This recommendation is further supported by case reports describing the use of vv-ECMO for conservative management following tracheal injury [27–29].

## Conclusion

Our case illustrates a rare and fatal presentation of serotonin syndrome and ARDS following multidrug and paroxetine poisoning, emphasizing the importance of early aggressive intervention and the limited efficacy of currently available extracorporeal elimination methods. Considering this clinical course as well as other reports of progressive symptom development over several days after paroxetine poisoning, clinicians should consider pharmacokinetic complications such as pharmacobezoars or poor CYP2D6 metabolism in massive SSRI overdose leading to an early and aggressive treatment in similar cases. This is specifically important as there are only limited options and evidence for interventions in serotonin syndrome as well as drug-induced ARDS, and in our case hemadsorption did not result in any noticeable paroxetine elimination.

## Supplementary Information

Supplementary Material 1. Video of inducible clonus

## Data Availability

The data that support the findings of this case report are available on request from the corresponding author. The data are not publicly available due to privacy or ethical restrictions.

## Abbreviations

ARDS: Acute respiratory distress syndrome
EEG: Electroencephalography
SEP: Somatosensory evoked potentials
SSRI: Selective serotonin reuptake inhibitor
vv-ECMO: Veno-venous extracorporeal membrane oxygenation

## References

[1] Chiew AL, Buckley NA. The serotonin toxidrome: shortfalls of current diagnostic criteria for related syndromes. Clin Toxicol (Phila). 2022;60(2):143–58. 34806513 10.1080/15563650.2021.1993242

[2] Chiew AL, Isbister GK. Management of serotonin syndrome (toxicity). Br J Clin Pharmacol. 2024. 10.1111/bcp.16152. 39343512 10.1111/bcp.16261

[3] Nelson LS, Erdman AR, Booze LL, Cobaugh DJ, Chyka PA, Woolf AD, *et al*. Selective serotonin reuptake inhibitor poisoning: an evidence-based consensus guideline for out-of-hospital management. Clin Toxicol (Phila). 2007;45(4):315–32. 17486478 10.1080/15563650701285289

[4] Jeong S, Kim Y, Choe S, Kang H, Kim HM, Kang JS. A fatal case of desvenlafaxine and paroxetine poisoning. J Pharm Biomed Anal. 2024;245: 116148. 38652939 10.1016/j.jpba.2024.116148

[5] Sato A, Okura Y, Minagawa S, Ohno Y, Fujita S, Kondo D, *et al*. Life-threatening serotonin syndrome in a patient with chronic heart failure and CYP2D6*1/*5. Mayo Clin Proc. 2004;79(11):1444–8. 15544025 10.4065/79.11.1444

[6] Canan F, Korkmaz U, Kocer E, Onder E, Yildirim S, Ataoglu A. Serotonin syndrome with paroxetine overdose: a case report. Prim Care Companion J Clin Psychiatry. 2008;10(2):165–7. 18458731 10.4088/pcc.v10n0213g PMC2292445

[7] Hudd TR, Blake CS, Rimola-Dejesus Y, Nguyen TT, Zaiken K. A case report of serotonin syndrome in a patient on selective serotonin reuptake inhibitor (SSRI) monotherapy. J Pharm Pract. 2020;33(2):206–12. 31030620 10.1177/0897190019841742

[8] Vermeulen T. Distribution of paroxetine in three postmortem cases. J Anal Toxicol. 1998;22(6):541–4. 9788532 10.1093/jat/22.6.541

[9] Prescott LF, Park J, Ballantyne A, Adriaenssens P, Proudfoot AT. Treatment of paracetamol (acetaminophen) poisoning with N-acetylcysteine. Lancet. 1977;2(8035):432–4. 70646 10.1016/s0140-6736(77)90612-2

[10] Force ADT, Ranieri VM, Rubenfeld GD, Thompson BT, Ferguson ND, Caldwell E, *et al*. Acute respiratory distress syndrome: the Berlin definition. JAMA. 2012;307(23):2526–33. 22797452 10.1001/jama.2012.5669

[11] Dunkley EJ, Isbister GK, Sibbritt D, Dawson AH, Whyte IM. The hunter serotonin toxicity criteria: simple and accurate diagnostic decision rules for serotonin toxicity. QJM. 2003;96(9):635–42. 12925718 10.1093/qjmed/hcg109

[12] Winek CL, Wahba WW, Winek CL Jr., Balzer TW. Drug and chemical blood-level data 2001. Forensic Sci Int. 2001;122(2–3):107–23. 11672964 10.1016/s0379-0738(01)00483-2

[13] Cooper J, Duffull SB, Isbister GK. Predicting serotonin toxicity in serotonin reuptake inhibitor overdose. Clin Toxicol (Phila). 2023;61(1):22–8. 36444913 10.1080/15563650.2022.2151455

[14] Li YK, Lam KF, Wong CLW, Wong A. In vitro study of pharmacobezoar formation in simulated acetaminophen overdose. Clin Toxicol (Phila). 2020;58(9):900–6. 31875726 10.1080/15563650.2019.1705971

[15] Kaye CM, Haddock RE, Langley PF, Mellows G, Tasker TC, Zussman BD, *et al*. A review of the metabolism and pharmacokinetics of paroxetine in man. Acta Psychiatr Scand Suppl. 1989;350:60–75. 2530793 10.1111/j.1600-0447.1989.tb07176.x

[16] Hilleret H, Voirol P, Bovier P, Giannakopoulos P, Zullino D, Baumann P, *et al*. Very long half-life of paroxetine following intoxication in an extensive cytochrome P4502D6 metabolizer. Ther Drug Monit. 2002;24(4):567–9. 12142644 10.1097/00007691-200208000-00017

[17] Damborska A, Hanakova L, Pindurova E, Horska K. Case report: therapeutic drug monitoring and CYP2D6 phenoconversion in a protracted paroxetine intoxication. Front Pharmacol. 2024;15:1444857. 39295933 10.3389/fphar.2024.1444857 PMC11408286

[18] Muzyk AJ, Jakel RJ, Preud’homme X. Serotonin syndrome after a massive overdose of controlled-release paroxetine. Psychosomatics. 2010;51(5):437–42. 20833944 10.1176/appi.psy.51.5.437

[19] Martin DC, Introna RP, Aronstam RS. Fentanyl and sufentanil inhibit agonist binding to 5-HT1A receptors in membranes from the rat brain. Neuropharmacology. 1991;30(4):323–7. 1830134 10.1016/0028-3908(91)90056-h

[20] Baldo BA, Rose MA. The anaesthetist, opioid analgesic drugs, and serotonin toxicity: a mechanistic and clinical review. Br J Anaesth. 2020;124(1):44–62. 31653394 10.1016/j.bja.2019.08.010

[21] Universitätsklinikums Erlangen (UKER). Stoffe, die CYP-Enzyme beeinflussen. https://kinderformularium.de/wechselwirkungen_CYP#CYP2D6.

[22] Ghannoum M, Hoffman RS, Gosselin S, Nolin TD, Lavergne V, Roberts DM. Use of extracorporeal treatments in the management of poisonings. Kidney Int. 2018;94(4):682–8. 29958694 10.1016/j.kint.2018.03.026

[23] Inoue F, Okazaki Y, Ichiba T, Kashiwa K, Namera A. Unexpectedly prolonged serotonin syndrome and fatal complications following a massive overdose of paroxetine controlled-release. Cureus. 2023;15(12): e50691. 38229825 10.7759/cureus.50691 PMC10791220

[24] Antonello N, Sergio L, Andrea T, Cornelius B. Pulmonary drug toxicity: presentation of a case of recurrent diffuse alveolar damage caused by paroxetine. Am J Ther. 2015;22(2):e43–7. 24284653 10.1097/MJT.0b013e31829ed1f2

[25] Coorey AN, Wenck DJ. Venlafaxine overdose. Med J Aust. 2019;1748(1):534. 10.5694/j.1326-5377.1998.tb141427.x 9631679

[26] Tonna JE, Abrams D, Brodie D, Greenwood JC, Rubio Mateo-Sidron JA, Usman A, *et al*. Management of adult patients supported with venovenous extracorporeal membrane oxygenation (VV ECMO): guideline from the Extracorporeal Life Support Organization (ELSO). ASAIO J. 2021;67(6):601–10. 33965970 10.1097/MAT.0000000000001432 PMC8315725

[27] Son BS, Cho WH, Kim CW, Cho HM, Kim SH, Lee SK, *et al*. Conservative extracorporeal membrane oxygenation treatment in a tracheal injury: a case report. J Cardiothorac Surg. 2015;10:48. 25885371 10.1186/s13019-015-0252-7 PMC4487840

[28] Sian K, McAllister B, Brady P. The use of extracorporeal membrane oxygenation therapy in the delayed surgical repair of a tracheal injury. Ann Thorac Surg. 2014;97(1):338–40. 24384192 10.1016/j.athoracsur.2013.04.126

[29] Buschulte K, Kahn N, Schmidt W, Reinhardt L. Severe tracheal tear–an alternative extracorporeal membrane oxygenation indication. Perfusion. 2024;39(6):1256–8. 37160714 10.1177/02676591231175983

